# ODin (Orthology Data driven Interaction) Predictor: A Client‑Side Web Application for Unveiling Biological Connections Through Orthology from Alliance of Genome Resources

**DOI:** 10.17912/micropub.biology.001923

**Published:** 2026-03-09

**Authors:** Jaehyoung Cho, Paul W Sternberg

**Affiliations:** 1 Division of Biology and Biological Engineering, California Institute of Technology, Pasadena, CA 91125, USA

## Abstract

Deciphering the complex landscape of gene interactions is essential for understanding biological systems and unraveling the mechanisms underlying disease. While experimental approaches to mapping these interactions are often labor-intensive and costly, computational strategies—particularly those that exploit evolutionary conservation through orthology—provide a powerful and scalable alternative. We present a novel client-side web application that infers gene interactions by leveraging comprehensive orthology and experimentally validated protein-protein or genetic interactions from the Alliance of Genome Resources. This tool substantially broadens known interactomes, especially for underrepresented model organisms such as zebrafish and *Xenopus* with less studied interactions. Its client-side architecture ensures exceptional data privacy and instantaneous results, setting it apart from conventional server-based platforms. By integrating these predictions with structural evidence and biological context, researchers may gain more reliable biological insights, helping to support more efficient and precisely targeted scientific discovery.

**Figure 1. Overview of the Client-Side Interaction Prediction Web Application and its Output:**
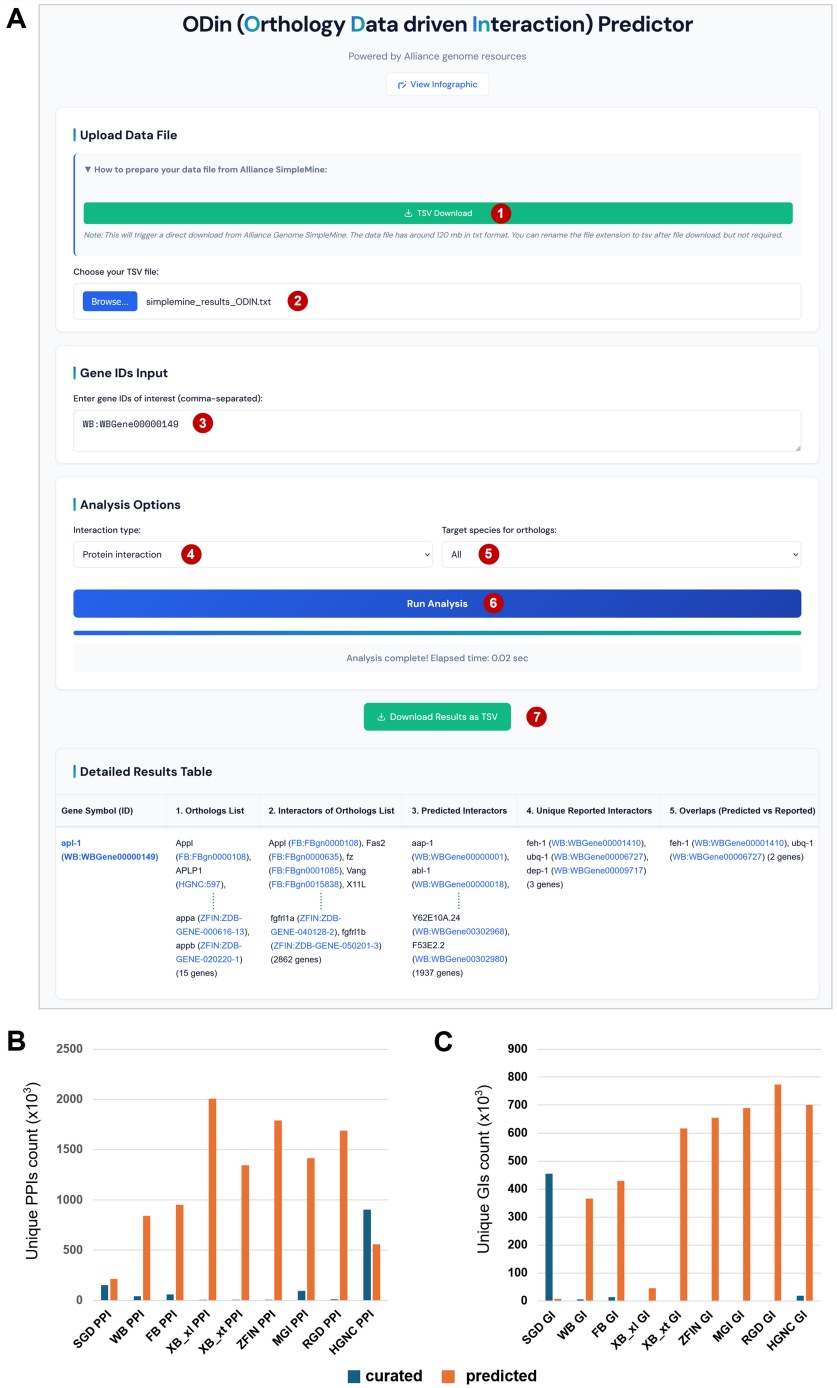
**(A) Web Application Front Page:** A screenshot illustrating the main user interface of the web application, showcasing the sections for downloading the Alliance SimpleMine data file, uploading the data file, inputting gene identifiers of interest, and selecting analysis parameters (interaction type and target species). The "Run Analysis" button initiates the predictive process. All essential steps are highlighted with red-circled numbers (step 1 to 7). The result table presents a comprehensive summary for each input gene, including its symbol and ID, the list of identified orthologs (filtered by selected species or all species), all annotated interactors of each orthologs, predicted interactors of these orthologs—known as ‘interologs’—that have been inferred for the target species, unique reported interactors of queried gene, and any overlaps between predicted and reported interactions. All gene IDs in the table are linked to their corresponding gene pages in the Alliance of Genome Resources. **(B) Comparison of Predicted vs. Curated Protein-Protein Interactions:** A bar graph illustrating the quantitative expansion of the interactome. This panel compares the number of protein-protein interactions newly predicted by the application against the number of previously reported protein-protein interactions for various species, demonstrating the added value of the orthology-based approach. (SGD: *Saccharomyces* Genome Database, WB: WormBase, FB: FlyBase, XB_xl: Xenbase *_ Xenopus laevis* , XB_xt: Xenbase _ *Xenopus tropicalis* . ZFIN: Zebrafish Information Network, MGI: Mouse Genome Informatics, RGD: Rat Genome Database, HGNC: HUGO Gene Nomenclature Committee) **(C) Comparison of Predicted vs. Curated Genetic Interactions:** A bar graph analogous to Panel B but specifically comparing the number of newly predicted genetic interactions against the number of previously reported genetic interactions for different species, highlighting the tool's capacity to uncover novel genetic associations.

## Description

Genes operate not in isolation, but as integral components of intricate networks encompassing diverse interactions—ranging from protein-protein interactions, which involve direct physical associations between gene products, to genetic interactions, where genes function in a common pathway (Sternberg and Huang, 2006; Szklarczyk et al., 2023). Mapping these interaction networks across species is essential for advancing biological understanding and identifying therapeutic targets. However, experimental approaches are often limited by substantial demands on time, cost, and technological resources. A persistent challenge stems from the prevailing research focus on well-conserved proteins within a narrow group of extensively studied model organisms. This bias inadvertently leads to significant gaps in interaction data for those same conserved genes in less-explored species, despite their recognized biological relevance, particularly in organisms that are less extensively studied such as zebrafish and *Xenopus* . Such omissions impede comprehensive systems-level analyses by leaving interaction networks underrepresented in key target organisms.

Orthologous genes, sharing a common ancestor through speciation, typically retain analogous functions and interactions across divergent species (Koonin, 2005; Matthews et al., 2001). This concept, foundational to "interolog" prediction (Matthews et al., 2001; von Mering et al., 2002), allows us to infer novel interactions from well-characterized ones. The Alliance of Genome Resources serves as a central, comprehensive data repository, providing high-quality, curated orthology assignments and integrated genetic and protein-protein interaction data from numerous model organisms (Alliance of Genome Resources Consortium, 2024). Harnessing data from the Alliance of Genome Resources for this predictive task offers several compelling advantages. The Alliance provides high-quality orthology relationships, both manually curated and computationally inferred, across a wide range of species, thereby ensuring robust gene connections that form a solid foundation for predictions (Alliance of Genome Resources Consortium, 2024). Furthermore, the platform consolidates diverse interaction data, encompassing both genetic and protein-protein interactions, from various validated sources, offering a rich repository of experimentally confirmed interactions for inferential purposes (Alliance of Genome Resources Consortium, 2024; Oughtred et al., 2021; Öztürk-Çolak et al., 2024; Porras et al., 2020; Sternberg et al., 2024). This facilitates cross-species knowledge transfer, enabling researchers to leverage the extensive biological knowledge accumulated in well-characterized model organisms to predict interactions in humans or other species where empirical data may be sparse. Consequently, this method aims to accelerate discovery by offering a rapid means to generate plausible hypotheses, thereby having the potential to guide experimental design and reduce the need for exhaustive, time-consuming, and expensive de novo experimental screens (Zhong and Sternberg, 2007).

&nbsp;

To streamline the exploration of interologs and the process of interaction prediction, a straightforward, browser-based web application has been developed and named ODin ( O rthology D ata driven in teraction) predictor (Extended data 1; https://github.com/WormBase/interolog_prediction). This tool enables researchers to input a list of gene identifiers and, utilizing a pre-downloaded data file from Alliance SimpleMine, predict orthologous genes and their potential interacting partners within a chosen target species. The analytical outcomes are presented in an easily interpretable, interactive table format and can be readily downloaded for subsequent in-depth analysis.

The complete analysis workflow consists of the following sequential steps, beginning with data acquisition and ending with the generation of a downloadable results table. All steps are highlighted in Figure 1A with red-circled numbers.

**Step 1 — Download the Required SimpleMine Data File**

- Open **ODin predictor.html** in a web browser.

- Click the **“Download SimpleMine TSV”** button provided on the web application’s interface.
- This button automatically sends a preset request to the Alliance SimpleMine service using predefined parameters.
- The request retrieves: Gene Symbol, Genetic Interaction, Protein‑Protein Interaction, All available Orthologs fields, Data for all gene from all species
- The complete dataset is downloaded directly to the user’s computer without requiring manual navigation through the SimpleMine website.
- Save the downloaded file as **simplemine_results.tsv** .

**Step 2 — Upload the Data File**

- Locate the file‑upload section.
- Click **“Choose File”** and select **simplemine_results.tsv** .
- Wait for the status message confirming successful loading and parsing.

**Step 3 — Enter Gene Identifiers**

- Input gene IDs with a comma separation into the text area.
- Ensure the identifiers match the format used in the uploaded SimpleMine file.

**Step 4 & 5 — Select Analysis Parameters**

- Choose the **interaction type** (protein‑protein or genetic).
- Select the **target species** for ortholog filtering.

**Step 6 — Run the Analysis**

- Click the **“Run Analysis”** button.
- Monitor the progress bar and status messages as the analysis executes.

**Step 7 — View and Download Results**

- After completion, the **Detailed Results Table** becomes visible.
- The table includes:
  - Gene symbol and identifier
  - Orthologs across species
  - Predicted interactors of orthologs
  - Interologs predicted for the target species
  - Experimentally reported interactors
  - Overlaps between predicted and reported interactions
  - Hyperlink to the gene ID to the corresponding gene page in Alliance of Genome Resources
- Click **“Download Results as TSV”** to save the full results table.

This tool offers a distinctive approach to gene interaction prediction. The strength lies in its exclusively client-side operation, which confers unparalleled data privacy and security and provides immediate results. Its direct integration with user-downloaded Alliance SimpleMine data is another salient feature. While established online resources such as STRING (Szklarczyk et al., 2023), BioGRID (Oughtred et al., 2021), and IntAct (Del Toro et al., 2022) focus on aggregating and visualizing *existing* empirical data, and other tools like the InteroPORC web server (Michaut et al., 2008) offer interolog prediction, our application precisely addresses the gap by empowering researchers to directly and locally leverage the rich, integrated orthology and interaction data from Alliance SimpleMine. This provides users with a high degree of autonomy over their source data, simplifying the workflow and enhancing the accessibility of computational prediction. The inherent power of this tool stems from its capacity for rapid hypothesis generation, significantly narrowing the experimental search space. This is particularly advantageous for model organisms like *Danio rerio* (zebrafish) and *Xenopus* species, which, despite their biological importance, often have fewer experimentally characterized interaction networks compared to extensively studied organisms like *Homo sapiens* or *Mus musculus* . For these species, our tool provides a crucial mechanism to rapidly expand their known interactomes, facilitating targeted functional studies and disease modeling where empirical data is currently limited. A comparative analysis of protein-protein interactions (PPIs) and genetic interactions (GIs) further underscores the value of this tool in quantitatively expanding the interactome coverage across diverse species. As demonstrated by the data (Figures 1C and 1D), predicted PPIs and GIs generally far exceed experimentally curated numbers. For instance, in *Danio rerio* (ZFIN), predicted PPIs were over 2,762 times greater than curated, and predicted GIs were 12,135 times more than curated GI. Similarly, other species also exhibit substantial increases in both predicted PPIs and GIs relative to their curated datasets—except for human PPIs and yeast GIs—highlighting the strong capacity of this approach to uncover novel potential interactions in species with limited experimental characterization. Overall, these findings highlight the immense value of orthology-based prediction in quantitatively enriching biological interaction networks, especially for less-studied model organisms, thereby having the potential to accelerate hypothesis generation and research in these areas. To further augment the robustness and practical utility of these computational predictions, future developments could involve incorporating the integration of complementary data types. For instance, predicted protein-protein interactions derived from orthology could be rigorously cross-referenced and validated using advanced structural prediction tools such as AlphaFold 3 (Abramson et al., 2024). AlphaFold 3's remarkable capability to accurately model protein structures, and increasingly, the structures of protein-protein complexes, offers a potent means to evaluate the physical plausibility of predicted interactions. Additionally, to discern the biological significance and functional context of these predicted interologs, their integration with Gene Ontology (GO) enrichment analysis (Ashburner et al., 2000) or pathway enrichment tools like Reactome (Milacic et al., 2024) would be invaluable. By identifying overrepresented biological processes, molecular functions, or cellular components, or by mapping them onto known metabolic or signaling pathways, researchers can prioritize interologs that are most functionally coherent and relevant to specific biological contexts. By merging interolog predictions with compelling structural evidence and biological contexts, researchers can significantly enhance confidence in the inferred interaction network, thereby prioritizing the most robust hypotheses for subsequent experimental validation. Such a synergistic integration would elevate preliminary predictions into highly reliable biological insights, fostering more efficient and precisely targeted scientific discovery.

## Methods

Technical Implementation

We designed and implemented the core logic, algorithms, and system architecture of the application, which operates entirely on the client side to ensure data privacy and deliver immediate responsiveness. To enable cross-platform compatibility, we employed Gemini 2.5 Flash to convert the original Python codebase into JavaScript, followed by front-end refinement and debugging to optimize usability and performance. Its architecture is built upon standard web technologies: HTML, CSS, and JavaScript. The user interface (UI) and structural layout are constructed using HTML5, providing a clear and intuitive environment for file uploads, parameter selection, and result presentation. Aesthetic design and responsive behavior across various device sizes are achieved through Cascading Style Sheets (CSS), which are embedded directly within the HTML document to ensure a clean and modern appearance.

All core application logic, encompassing data handling, computational analysis, and dynamic UI updates, is implemented in JavaScript. Upon file upload, the application performs in-browser parsing of the tab-separated values (TSV) data. This parsing process specifically bypasses the initial header rows of the Alliance SimpleMine file and efficiently stores the pertinent gene information, including Gene ID, Gene Symbol, Genetic Interactions, Protein Interactions, and Orthology data, into an efficient in-memory JavaScript object (hash map). Gene IDs serve as keys, facilitating rapid data retrieval. The analytical pipeline executed by JavaScript includes several key steps. First, it processes user-provided gene IDs from the text area, cleaning and parsing them for subsequent analysis. For each input gene, the application identifies its orthologs based on the loaded Alliance data. This step initially retrieves all known orthologs and subsequently filters them according to the user's selected target species. Following this step, the application identifies known interactors (either genetic or protein) for these species-filtered orthologs from the in-memory dataset. Interolog prediction is then performed by mapping the interactors of orthologs back to the target species. Finally, the application compares these predicted interologs with any experimentally reported interactions for the original input gene within the target species, highlighting any overlaps. All derived results are dynamically rendered into an HTML table for user review and are available for export as a TSV file.

A pivotal advantage of this web application is its entirely client-side operation, meaning all data processing occurs directly within the user's web browser. This design choice inherently enhances data privacy and security, as sensitive research data is never transmitted to or stored on an external server. It also provides immediate results without the latency associated with server communication or queuing, offering a truly private and responsive user experience.

&nbsp;

Genome-Wide Data Acquisition and Interaction Analysis

All necessary data for genome-wide interaction prediction were obtained from the SimpleMine tool (https://www.alliancegenome.org/agr_simplemine.cgi) at Alliance of Genome Resources ( https://www.alliancegenome.org , ver 8.2.0). Unique curated and predicted molecular or genetic interactions were parsed from the original data file and analyzed using a Python (ver. 3.13.7) script. The resulting comparisons were visualized using Microsoft Excel (Microsoft 365, ver. 2507).

## Data Availability

Description: ODin interolog prediction web application version 1.1. Resource Type: Software. DOI: https://doi.org/10.22002/b9z2t-7g394
